# Current and Future Approaches for Diagnosing Small Intestinal Dysbiosis in Patients With Symptoms of Functional Dyspepsia

**DOI:** 10.3389/fnins.2022.830356

**Published:** 2022-05-06

**Authors:** Ayesha Shah, Nicholas J. Talley, Gerald Holtmann

**Affiliations:** ^1^Faculty of Medicine and Faculty of Health and Behavioural Sciences, The University of Queensland, Brisbane, QLD, Australia; ^2^Department of Gastroenterology and Hepatology, Princess Alexandra Hospital, Brisbane, QLD, Australia; ^3^AGIRA (Australian Gastrointestinal Research Alliance) and the NHMRC Centre of Research Excellence in Digestive Health, Newcastle, NSW, Australia; ^4^College of Health, Medicine and Wellbeing, University of Newcastle, Callaghan, NSW, Australia

**Keywords:** small intestinal dysbiosis, small intestinal bacterial overgrowth, functional dyspepsia, functional gastrointestinal disorders, breath tests, small bowel aspirate and culture

## Abstract

The development and application of next generation sequencing technologies for clinical gastroenterology research has provided evidence that microbial dysbiosis is of relevance for the pathogenesis of gastrointestinal and extra-intestinal diseases. Microbial dysbiosis is characterized as alterations of diversity, function, and density of the intestinal microbes. Emerging evidence suggests that alterations of the gastrointestinal microbiome are important for the pathophysiology of a variety of functional gastrointestinal conditions, e.g., irritable bowel syndrome (IBS) and functional dyspepsia (FD), also known as disorders of brain-gut axis interaction. Clinicians have for many years recognized that small intestinal bacterial overgrowth (SIBO) is typified by a microbial dysbiosis that is underpinned by abnormal bacterial loads in these sites. SIBO presents with symptoms which overlap with symptoms of FD and IBS, point toward the possibility that SIBO is either the cause or the consequence of functional gastrointestinal disorders (FGIDs). More recently, new terms including “intestinal methanogen overgrowth” and “small intestinal fungal overgrowth” have been introduced to emphasize the contribution of methane production by archea and fungi in small intestinal dysbiosis. There is emerging data that targeted antimicrobial treatment of SIBO in patients with FD who simultaneously may or may not have IBS, results in symptom improvement and normalization of positive breath tests. However, the association between SIBO and FGIDs remains controversial, since widely accepted diagnostic tests for SIBO are lacking. Culture of jejunal fluid aspirate has been proposed as the “traditional gold standard” for establishing the diagnosis of SIBO. Utilizing jejunal fluid culture, the results can potentially be affected by cross contamination from oropharyngeal and luminal microbes, and there is controversy regarding the best cut off values for SIBO diagnosis. Thus, it is rarely used in routine clinical settings. These limitations have led to the development of breath tests, which when compared with the “traditional gold standard,” have sub-optimal sensitivity and specificity for SIBO diagnosis. With newer diagnostic approaches–based upon applications of the molecular techniques there is an opportunity to characterize the duodenal and colonic mucosa associated microbiome and associated gut microbiota dysbiosis in patients with various gastrointestinal and extraintestinal diseases. Furthermore, the role of confounders like psychological co-morbidities, medications, dietary practices, and environmental factors on the gastrointestinal microbiome in health and disease also needs to be explored.

## Introduction

It is now well established that the human microbiome hosts trillions of microorganisms. These include bacteria, viruses, bacteriophages, and fungi (Sender et al., 2016). The microbes colonizing the human gastrointestinal tract are mainly composed of bacteria but also includes archea, viruses, fungi, and protozoa. These microbes are important for key functions of the gastrointestinal tract -digestion of food, production of vitamins, absorption of micronutrients (Resta, 2009) immune hemostasis (Wu and Wu, 2012). maintaining gut barrier function (Camilleri et al., 2012), and protection against pathogens (Quigley, 2013). In recent years, culture independent techniques have enabled identification and characterization of the microbes that colonize all segments of the human gastrointestinal tract (Bik et al., 2006). Segments of the gastrointestinal tract such as the duodenum that were conventionally believed to be “sterile”–due to an unfavorable environment with an acidic pH in the stomach or high proteolytic activity of aggressive pancreatic enzymes and bile acids (Guarner and Malagelada, 2003) (duodenum)–harbor microbes that are adapted to the harsh environment. Thus, a large number of factors including the milieu, availability of specific nutritional factors and medications will influence the composition of the luminal and likely the mucosa associated microbiome. Next-generation sequencing utilizing deep, high-throughput, in-parallel DNA sequencing technologies have revealed deep insights into the taxonomic and functional diversity of the intestinal microbiota linked to gut physiology and its potential role in the pathophysiology of various diseases (Loman and Pallen, 2015).

Composition, function (including the metabolic properties) and density of the microbes colonizing the mucosal lining of the gastrointestinal tract collectively define the gastrointestinal microbiome. It is now acknowledged that soon after birth, the gastrointestinal tract is colonized by microbes from the environment (e.g., family members) (Dominguez-Bello et al., 2010). The density of bacteria in the human gastrointestinal tract shows an increase continuously from 10^1^ to 10^3^ bacteria cfu/ml in the stomach and duodenum, toward 10^4^ to 10^7^ bacteria cfu/ml in the distal small intestine and reaching 10^11^ to 10^12^ cfu/ml in the colon (O’Hara and Shanahan, 2006). Furthermore, there are distinct differences in the microbes in the intestinal lumen as compared to microbes in the mucus layer and the proximity of the intestinal epithelium (Swidsinski et al., 2005; Sekirov et al., 2010; Shanahan et al., 2016). The microflora of the stomach is gram-positive aerobes, fungi and facultative anaerobes, mirroring the microflora in the oropharynx (Gorbach et al., 1967). It is evident that the duodenum and small intestine represent a transition zone between the sparsely populated stomach and the high-density bacterial flora of the colon. While the mid-distal small intestine and colon are represented predominantly by gram-negative bacteria and anaerobes (Gorbach et al., 1967; Drasar and Shiner, 1969; Drasar et al., 1969). The differences in the concentration and type of bacteria along the different segments of the gastrointestinal tract can be largely explained by the microenvironment and anatomical differences along the gastrointestinal tract.

Relevant changes in the diversity, density or metabolic function of gut microbes is frequently referred to as intestinal “dysbiosis” although the exact cutoff thresholds are poorly defined (Shah et al., 2018). There is accumulating evidence that intestinal dysbiosis is associated with the conditions such as functional dyspepsia (FD) (Gurusamy et al., 2021), irritable bowel syndrome (IBS) (Shah et al., 2020a), inflammatory bowel disease (IBD) (Gandhi et al., 2021), celiac disease (Losurdo et al., 2017), or even several extra-intestinal disorders (Carding et al., 2015; Lynch and Pedersen, 2016). The most widely recognized and studied small intestinal dysbiosis is small intestinal bacterial overgrowth (SIBO). SIBO is a clinical disorder, defined by an abnormal microbial density (loads) and/or abnormal types of microbes in these sites (Corazza et al., 1990; Bouhnik et al., 1999). Many studies point toward the fact that the contaminating flora seen in SIBO has features of microbes typically found in the oropharyngeal space and/or the colon (Bouhnik et al., 1999). Gastrointestinal symptoms that are considered typical for SIBO include diarrhea, fullness, bloating, flatulence, abdominal pain and discomfort, or weight loss (Grace et al., 2013) and SIBO can result in structural changes including villus atrophy (Riordan et al., 2001) and malabsorption. SIBO may occur simultaneously with other gastrointestinal disorders and with relatively unspecific symptoms it is often challenging to determine if SIBO is simply the cause or the result in relation to the other disorder (Ghoshal et al., 2003; Quigley and Abu-Shanab, 2010). In this review article we aim to (a) describe the link between small intestinal dysbiosis and FD and (b) critically appraise currently available and future diagnostic approaches to characterize small intestinal dysbiosis.

## Conceptional Framework for Functional Dyspepsia and Relation to Other Functional Gastrointestinal Disorders

The term functional dyspepsia (FD) is used for patients presenting with recurring symptoms of indigestion referred to upper gastrointestinal tract without obvious identifiable cause for these symptoms (Enck et al., 2017; Holtmann et al., 2017). Many patients presenting with FD report concomitant symptoms of IBS and both conditions are frequently found in patients with more severe symptoms (von Wulffen et al., 2019). While both conditions are associated with more or less specific gastrointestinal symptoms, with no structural or biochemical abnormalities explaining these symptoms, the concept of a FD or IBS manifesting without defined pathophysiology is long outdated. With appropriate diagnostic tests, subtle structural or biochemical abnormalities can be found in many patients with FD and/or IBS that may explain the symptoms. These factors include intolerance of specific dietary components, chronic infections, changes in the gastrointestinal microbiota, minimal mucosal inflammation with subtle increase and degranulation of eosinophils and mast cells, systemic activation of the immune system, changes in the integrity of the intestinal barrier and subsequent intestinal permeability, altered bile salt metabolism, abnormalities in the serotonin metabolism and genetic factors (Holtmann et al., 2017). Thus far most research in relation to SIBO has focused on IBS and it is reasonable to assume that many if not the majority of patients included in these studies focusing on IBS also had FD.

## Small Intestinal Dysbiosis and Functional Dyspepsia

The role of the human microbiome in regulating physiological functions including gastrointestinal motor function, gastric and pancreatic secretion, protection of the epithelial barrier, and the interplay between the gut and the central nervous system potentially explains its contribution to symptoms associated with FGID (Saffouri et al., 2019). Recent systematic reviews and meta-analyses have confirmed a link between SIBO and both IBS (Shah et al., 2020a) and FD (Gurusamy et al., 2021), the two most common functional gastrointestinal disorders (FGIDs). In patients with FD, there is a significantly increased (OR 4.3, 95% CI 1.1–17.5) prevalence of SIBO diagnosed utilizing breath tests, compared to healthy controls, with no significant difference in SIBO prevalence according to FD sub-types; namely, epigastric pain syndrome (EPS) and post prandial distress syndrome (PDS) (Gurusamy et al., 2021).

Small intestinal bacterial overgrowth theoretically may also play a critical role for the pathogenic mechanism that characterizes FD occurring after a gastrointestinal infection, referred to as post-infectious FD. Post-infectious functional dyspepsia is a clinical entity that manifests after an episode of acute gastroenteritis [commonly induced by *Salmonella* spp., *Escherichia coli O157, Campylobacter jejuni, Giardia lamblia*, and *Norovirus* (Futagami et al., 2015)]. Post-infectious functional dyspepsia occurs in one out of 10 individuals, and the estimated odds ratio is 2.5 at 6 months following an episode of acute gastroenteritis as compared to controls within the same population (Futagami et al., 2015). To the best of our knowledge none of the studies have assessed the prevalence of SIBO in patients with post-infectious FD.

### Role of Methanogens

There has been emerging data regarding the role of methane and its effect on the gut function. In humans, *Methanobrevibacter smithii* (Eckburg et al., 2005) is a predominant methanogen, and principally rely on the production of methane from hydrogen (H_2_) and carbon dioxide (CO_2_) as their only source of energy (Sahakian et al., 2010). Thirty-six to 50% of healthy adult subjects are predominantly methane producers (Levitt et al., 2006). There has been growing attention regarding the link between methane production and constipation (Kunkel et al., 2011; Gandhi et al., 2021 in both patients with IBS and functional constipation, since methane could inhibit motility and slow gastrointestinal transit (Kunkel et al., 2011; Ghoshal et al., 2018). Thus, SIBO could lead to symptoms associated with FD *via* a delay of small intestinal motility and transit. Indeed, a recent systematic review and meta-analysis observed an association between FD and SIBO (Gurusamy et al., 2021). However, the data are very limited in relation to the pathophysiologic role of methane positive SIBO in FD.

### Small Intestinal Fungal Overgrowth

In humans, the number of microbial cells (including bacteria, archea, viruses, and fungi) by far exceeds the number of host cells. However, the majority of studies characterizing the gastrointestinal microbiome focus on characterizing the “bacterial colonization” of the gastrointestinal tract, and very limited data are available in relation to fungi and viruses. Fungus, and specifically *Candida* species, colonizes the gut (Nobile and Johnson, 2015). Fungal overgrowth in the small intestine can potentially cause otherwise unexplained gastrointestinal symptoms. Small intestinal fungal overgrowth (SIFO) is defined by the presence of excessive amounts of fungi in the small bowel and related to unexplained gastrointestinal symptoms. The relevance of fungal growth in small intestine has not been appropriately evaluated in patients with unexplained gastrointestinal symptoms (Erdogan and Rao, 2015). Jacobs et al. (2013) found that in 150 patients with unexplained gastrointestinal symptoms and negative endoscopy, 24/150 had SIFO and 32/150 had mixed SIFO/SIBO when their duodenal fluid was aspirated and cultured. Streptococcus, Enterococcus, Klebsiella and E. coli were predominantly found in SIBO while SIFO was linked to Candida. They also found that while gastrointestinal symptom profiles were not different between subjects with or without SIBO/SIFO, small intestinal dysmotility and proton pump inhibitor (PPI) use were identified as risk factors for SIBO or SIFO. Subsequently Erdogan et al. (2014) focused on the prevalence of SIFO in patients with unexplained gastrointestinal symptoms, and observed that 25.3% of a consecutive patients had SIFO. SIFO potentially may cause symptoms such as nausea, belching, gas, bloating, indigestion, and diarrhea, but all these symptoms overlap with those of SIBO and FGIDs. Thus, further case-control studies are warranted to further define the links between SIFO and FD and most importantly the role of treatments targeting SIFO.

### Effects of Treatment With Proton Pump Inhibitor on Small Intestine Bacterial Overgrowth and Functional Dyspepsia

Proton pump inhibitor are frequently used for the treatment of patients with FD (Pinto-Sanchez et al., 2017). PPI increases the gastric pH, and this increases the risk of gastrointestinal infections by weakening the barrier for microbial infections provided by the acidic gastric milieu (Brophy et al., 2013). It is also now well recognized that PPI use has effects on the fecal microbiome (Imhann et al., 2016). Imhann et al., found PPI use to be linked to decreased bacterial diversity and changes in 20% of the bacterial taxa (Imhann et al., 2016). In addition, they found that in PPI users oral bacteria and potentially pathogenic bacteria were over-represented in the fecal microbiome. This includes the genera *Rothia, Enterococcus, Streptococcus, Staphylococcus*, or the potentially pathogenic species *E. coli*. While there has been considerable data on stool microbiome (which might be of limited relevance), much less information is available in relation to the link between PPI use and small intestinal bacterial colonization or even the mucosa associated microbiome. In a recent study by Weitsman et al. (2022), SIBO rates were not significantly different between subjects on a PPI as compared to those not on a PPI, based upon duodenal aspirate and culture (>10^3^ CFU/ml) or 16S sequencing. However, a recent systematic review and meta-analysis (Gurusamy et al., 2021), found SIBO prevalence higher in FD patients on a PPI (66.7%, 95% CI 38.4–88.2) as compared to FD patients not on a PPI (27.8%, 95% CI 9.7–53.5), however, this just failed statistical significance. Furthermore, utilizing quantitative polymerase chain reaction (qPCR) (Shah et al., 2020b), 56 PPI users had a significantly increased bacterial load in the duodenum compared to 181 non-PPI users.

### Symptoms of Functional Dyspepsia and Small Intestinal Dysbiosis

**FIGURE 1:**
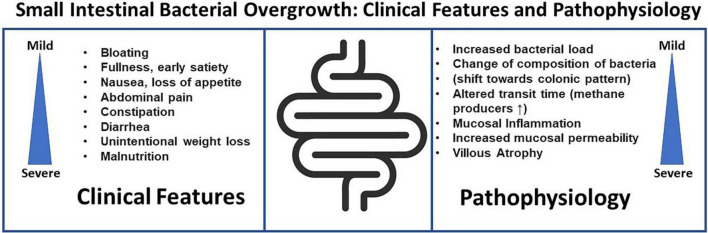
Small intestinal bacterial overgrowth, clinical features and pathophysiology.

Gastrointestinal symptoms found in association with SIBO include bloating, abdominal distension, flatulence, abdominal discomfort–but many of these are also symptoms of FD. Although the link between SIBO and FD or more broadly FGID is well acknowledged, it remains to be established if SIBO is indeed causing the gastrointestinal symptoms, the consequence of an underlying abnormality of gut function or an epiphenomenon in relation to FGIDs. Clinical symptoms in patients with SIBO are variable. A recently published elegant review by Grace et al. (2013), found diarrhea to be the most common SIBO symptom followed by abdominal pain and bloating. However, while a variety of symptoms have linked to SIBO, validated questionnaires for symptom assessment have been rarely used. Signs of severe SIBO may include nutrient malabsorption (Gutierrez et al., 2012), with weight loss, steatorrhea, and decreased levels of fat-soluble vitamins, and deficiencies of vitamin B12, folate, and iron. Contrasting these data suggesting a link between SIBO or SIFO and specific symptoms, Jacobs et al. (2013) could not verify a link between a specific symptom or cluster of symptoms with either SIBO or SIFO. Thus, symptoms remain at best generally poor predictors of bacterial and/or fungal overgrowth, hence testing is essential (Figure 1).

Quigley (2014) introduced the concept of “Classical SIBO” and “SIBO in FGIDs.” In “classical SIBO” clinical features can be pathophysiologically explained by the changes in the morphology of the small intestine. This may refer to SIBO in patients with “stagnant loop syndrome” who present with atrophy of intestinal villi and subsequent symptoms of maldigestion and malabsorption. In this traditional concept of SIBO, jejunal fluid culture remains an important diagnostic test as abnormal results correlate with severity of the clinical manifestation. In the more recent concept of “SIBO in FGIDs,” unexplained gastrointestinal symptoms are linked to SIBO without maldigestion and/or malabsorption. The precise pathophysiology and the mechanisms remained to be established to ensure that SIBO is truly the cause for FGIDs, and not SIBO caused by the underlying pathophysiology of FGID or an epiphenomenon (Quigley, 2014).

## Characterization of the Small Intestinal Microbial Colonization

### Current Diagnostic Approaches to Characterize Bacterial Colonization of the Small Intestine

#### Small Bowel Aspirate and Culture

So far aspiration of small bowel fluid and subsequent culture of the aspirate has been regarded as the “gold standard” for studying small intestinal microflora and establishing the diagnosis of SIBO (Corazza et al., 1990). Traditionally, confirming ≥10^5^ colony forming units per milliliter (cfu/ml) of colonic-type bacteria in the culture of the small bowel aspirate is considered as the established criteria for diagnosing SIBO (Bardhan et al., 1992; Paik et al., 2011). However, these diagnostic thresholds have been challenged, largely because this cut-off value was established from samples obtained from patients who typically have altered post-surgical gastrointestinal tract anatomy such as surgical removal of the ileocolonic valve, or presence of entero-enteral fistula or “blind loop syndrome” following surgical diversion (Bardhan et al., 1992; Paik et al., 2011). A landmark review by Khoshini et al. (2008), revealed that the small intestinal bacterial counts were significant lower at 0–10^3^ cfu/ml in healthy asymptomatic adults. This work and further studies have questioned the appropriateness of ≥10^5^ cfu/ml as cut-off threshold for SIBO diagnosis (Khoshini et al., 2008; Jacobs et al., 2013; Erdogan and Rao, 2015). Thus, a bacterial concentration of ≥10^3^ cfu/ml is now frequently considered as a new threshold (Pyleris et al., 2012; Jacobs et al., 2013) for diagnosing SIBO. This is clinically relevant as luminal aspirates are frequently obtained from the duodenum using a standard gastroscope despite using the same diagnostic thresholds for bacterial counts (Bohm et al., 2013). Indeed, a cut off threshold of ≥10^3^ cfu/ml may be appropriate for aspirations obtained from the duodenum given its proximal location, relative protection from translocation of bacteria from the colon and its frequent exposure to acid from stomach, all of which would decrease risk of SIBO (Jacobs et al., 2013; Walker and Talley, 2014; Erdogan and Rao, 2015). Further studies are still required to define the normal level of bacteria (and other microbes) in small bowel in the healthy controls supporting a more accurate definition of SIBO.

Independent of the above considerations, there are several other significant limitations with culture techniques for the diagnosis of SIBO. Sampling of small intestinal fluid can be achieved during endoscopy, utilizing small intestinal tubes positioned fluoroscopically, capsule biopsy or intraoperative bowel aspirate (Plaut et al., 1967). However, the optimal technique has not yet been defined (Corazza et al., 1990; Bardhan et al., 1992; Leon-Barua et al., 1993). In case of SIBO due to obligate anaerobes the culture results might be falsely negative (Tabaqchali, 1970). Furthermore, culture results may be falsely negative if intestinal bacterial overgrowth is patchy and only affects specific segments of the upper gastrointestinal tract (Tillman et al., 1981). Moreover, currently available methods do not allow sampling of the lower gastrointestinal tract, hence distal bacterial overgrowth can be overlooked (Corazza et al., 1990; Lin, 2004; Fan and Sellin, 2009).

One of the concerns in SIBO diagnosis is the potential risk of contamination of the small bowel aspirate by microbes colonizing the oropharynx during intubation of the gastrointestinal tract *via* the mouth and this may result in false positives and an overrating of SIBO prevalence (Hamilton et al., 1982). Despite various approaches to avoid cross contamination during intubation (i.e., use of oral antiseptics, simultaneous culture of saliva and aspirate) (Hamilton et al., 1982), the use of sterile gloves, and positioning a sterile catheter in the small intestine, the risks cannot be completely eliminated (Cangemi et al., 2021). More recently, our group has developed an aseptic biopsy device (the Brisbane Aseptic Biopsy Device, BABD) which allows mucosal biopsies to be obtained from the gastrointestinal tract that are not cross contaminated by oral or luminal contents (Zhong et al., 2017).

During endoscopy sampling of the small intestinal fluid can be technically challenging as frequently only small quantities of fluid that can be readily aspirated. In addition, aspiration may extend the required procedure time (Riordan et al., 1995). To overcome this limitation, an alternate approach would be culturing biopsies obtained endoscopically from the small intestine. Microbes are found in the mucus layer, covering the intestinal epithelium; thus biopsies taken during endoscopy and culture of these biopsy samples can be a fast and an efficient approach compared to aspiration in order to study the small intestinal microflora (Plaut et al., 1967). Indeed, the currently available two studies have reported significant correlations between culture of mucosal biopsies and aspirate for the diagnosis of SIBO. In addition, these studies found significant correlations with regards to the total bacterial counts, type of organisms (Riordan et al., 1995) and presence or absence of SIBO (Chandra et al., 2010). This suggests that culture of mucosal biopsies can be a potential alternative to the so far accepted gold standard of small bowel aspirate and culture for the diagnosis of SIBO.

#### Breath Tests

Breath tests, based on the measurement of hydrogen (H_2_) and methane (CH_4_) gas in breath samples are the most widely used test for diagnosing SIBO (Khoshini et al., 2008). Mammalian cells do not produce H_2_ or CH4 (Levitt, 1969). Breath tests are based on the principal, that metabolism of carbohydrate residue by gastrointestinal bacteria leads to the production of H_2_ and/or CH_4_ gas, which can be measured in exhaled breath sample. Thus, H_2_ or CH_4_ in the human breath indicates presence of gut bacteria that metabolize (otherwise non-fermentable) carbohydrates in the gut (Simrén and Stotzer, 2006). After oral ingestion of glucose and lactulose, H_2_ and CH_4_ are quantified as parts per million (ppm) in the exhaled breath using, e.g., gas chromatography (Christman and Hamilton, 1982). Breath tests which are indirect tests have largely replaced small bowel aspirate and culture in the clinical setting for diagnosing SIBO. The most recent consensus on breath testing for SIBO (Rezaie et al., 2017), defines a rise over baseline of ≥20 parts per million (ppm) for H_2_ within 90 min (after ingestion of the carbohydrate glucose or lactulose) or a level of ≥10 ppm of CH_4_ is considered a positive test to diagnose SIBO.

More than thirty percent of healthy adult subjects are predominantly methane producers (Bjorneklett and Jenssen, 1982). Therefore, if only hydrogen is analyzed during breath testing, the results may be falsely negative in a significant proportion of subjects. Hence the recent American College guidelines have coined the term “intestinal methanogen overgrowth (IMO),” for emphasizing the importance of methane production by methanogens belonging to the domain archea rather than intestinal dysbiosis driven solely by bacteria (Pimentel et al., 2020)The two most used breath tests are glucose breath test (GBT) and lactulose breath test (LBT).

##### Glucose Breath Test

In the year 1976 GBT was developed for the assessment of SIBO (Metz et al., 1976). The substrate glucose is rapidly absorbed in the proximal small bowel, and thus rarely reaches the colon (Bond and Levitt, 1972; Sellin and Hart, 1992) making it an attractive substrate to detect at least proximal SIBO. If the GBT is negative, this does not rule out SIBO that affects primarily the distal small bowel. Therefore, GBT favors specificity over sensitivity (Saad and Chey, 2014; Pimentel, 2016).

##### Lactulose Breath Test

The rise of H_2_ levels following ingestion of lactulose was first described by Bond and Levitt (1972). Contrasting glucose, lactulose is not absorbed in the small intestine and consequently metabolized by colonic bacteria. This bacterial metabolism of lactulose results in a production of H_2_ and/or CH_4_. Thus lactulose breath testing can be used–apart from detecting SIBO–also to measure the orocaecal transit time (Hirakawa et al., 1988). While lactulose remains in the lumen of the small intestine, it theoretically should also be able to detect bacteria colonizing the distal small intestine (Rhodes et al., 1979). This means that unlike GBT, LBT favors sensitivity over specificity. Moreover, there are concerns that LBT measures oro-cecal transit and not SIBO. A recent study in IBS patients, simultaneously measuring oro-cecal transit time (using^99*m*^Tc scintigraphy) and SIBO using LBT, found that the abnormal rise in H_2_ measured in the LHBT could be due to variations in oro-cecal transit time and not necessarily due to SIBO (Yu et al., 2011). Furthermore, there are multiple cut-off thresholds, without adequate validation or consensus for diagnosing SIBO utilizing a LBT. The most common criteria used for SIBO diagnosis utilizing LBT is the “double peak effect.” The detection of two distinguishable H_2_ peaks is not a reliable criterion, as it has been shown in transit studies that a bolus can reach the caecum, imitating the first peak followed by the body of lumen contents, producing a second peak, yielding false positive results (Riordan et al., 1996; Sadik et al., 2003). Several systematic reviews and meta-analyses (Shah et al., 2020a; Gurusamy et al., 2021) assessing SIBO prevalence in various gastrointestinal disorders and recent North American consensus statement and guidelines (Rezaie et al., 2017) have raised concerns that LBT (as compared to other modalities for diagnosing SIBO) overestimates SIBO prevalence rates in both cases and controls.

##### Limitations of Breath Tests

There are several limitations to breath tests as diagnostic test for SIBO. Overall, the sensitivity and specificity for breath test is poor and there is poor correlation with aspiration and culture method. As compared to the aspiration and culture method, the GBT has a sensitivity of 62.5% and a specificity of 81.7%, while the LBT has a sensitivity of 52.4% and a specificity of 85.7% (Gasbarrini et al., 2009). There is no agreement on the optimal substrate, the best doses of substrates, the duration of the test, time interval between samples or even the definition of a normal and abnormal breath test (Shah et al., 2018). Notably in patients with SIBO gas production is variable and is clearly dependent on the concentration and the type of colonizing bacteria in the small bowel, H_2_ or CH_4_ predominant overgrowth, absorptive capacity of the small bowel and availabilities of carbohydrate residues. The potential causes for false positive or false negative breath test are summarized in Table 1.

**TABLE 1:** Causes for false positive and false negative indirect tests for small intestinal bacterial overgrowth (SIBO).

False positive breath test	False negative breath test
Carbohydrate malabsorption (chronic pancreatitis and coeliac disease).	Hydrogen sulfide production by certain bacteria “consuming” hydrogen or Archea, metabolizing hydrogen and producing methane can cause low hydrogen levels resulting in false negative breath tests if only hydrogen is measured.
Gastrointestinal disorders with rapid gastric emptying.	Gastrointestinal disorders with delayed gastric emptying.
Oral bacterial flora.	Exercise prior to the test.
Smoking.	Antibiotic therapy.
High fiber diet, leaving residue of poorly absorbable carbohydrates in the colon, with subsequent colonic fermentation and gas production.	

### Newer Diagnostic Approaches

#### Molecular Assays

Culture-based methods have obvious limitations. In particular they do not allow to appropriately determine the microbial diversity of the intestinal microbiota since a large fraction of the microbiota cannot be cultured (Eckburg et al., 2005). Indeed, in humans recent studies that applied a variety of culture independent molecular assays demonstrate a thus far unrecognized complexity of the gut microbes with large numbers of phylotypes, of which 80% have so far not been cultured (Zoetendal et al., 2008). A large number of studies have revealed gut microbial dysbiosis based upon compositional changes in the microbial communities, principally *via* taxonomy-based assessments using 16S rRNA gene amplicon sequencing. More recently, shotgun metagenomic sequencing have been applied to fecal samples and similar technologies have been applied to study the bacterial genome (metagenomics), expressed mRNA in gastrointestinal tract samples (metatranscriptomics), produced proteins (metaproteomics), and the metabolite profiles (metabolomics). Metagenomics assess genes that could be expressed, while metatranscriptomics focuses on regulatory networks or gene expression and combined with metaproteomics, and metabolomics evaluates the functionality of the microbiota and, therefore, supports work to gain a better understanding of microbial activities in the gut and the potential effects for the host.

While these approaches have provided huge steps into a better understanding of the role of microbes for gut physiology key knowledge gaps remain. Thus far most work has focused on the fecal microbiota simply because fecal samples are easy to obtain. On the other hand, the stool microbiome is largely influenced by the intestinal/colonic transit and diet (Daniel, 2022). Consequently, stool may not be representative of microbes colonizing the mucosa usually addressed as the mucosa associated microbiome (MAM) (Gorkiewicz et al., 2013; Gevers et al., 2014; Li et al., 2015; Sundin et al., 2015; Leite et al., 2020). In this context, in a pilot study of patients diagnosed with FD, a qPCR approach targeting the 16S rRNA gene from Domain Bacteria and the human beta-actin gene was used to evaluate the efficacy of different DNA extraction methods to recover MAM (Cuiv et al., 2011); and later to objectively measure duodenal mucosal bacterial load. The bacterial load on duodenal tissue samples in nine FD patients showed a strong inverse correlation with patient quality of life (QoL) scores, and strong positive correlation with symptom severity following a nutrient challenge test (NCT) (Shanahan et al., 2016; Zhong et al., 2017). Thus, qPCR offers a novel culture-independent assessment of bacterial load on small intestinal tissue, to objectively identify SIBO. Patients with functional dyspepsia also were found to have a greater relative abundance of Streptococcus and decreases in the relative abundance of other genera such as *Prevotella, Veillonella*, and *Actinomyces* compared with control subjects, suggesting that their symptoms may be related to alterations of their microbiome at this site. The findings of the pilot studies (Shanahan et al., 2016, 2018; Zhong et al., 2017). now have been replicated in a larger cohort of patients with FGID, IBD in remission and asymptomatic controls (Shah et al., 2020b). Duodenal bacterial load was significantly higher in FGID patients as compared to asymptomatic controls with negative endoscopy results, and those with IBD in remission, and this was independent of PPI use. On the other hand, there was no significant differences in the prevalence of abnormal glucose breath testing across the different patient groups and controls.

Another recent elegant study by Leite et al. (2020), compared the duodenal microbiome composition in SIBO and non-SIBO subjects, using a cut off threshold of ≥10^3^ CFU/ml for diagnosing SIBO from duodenal aspirates. 16S ribosomal RNA (rRNA) sequencing revealed that SIBO subjects had 4-fold significantly higher relative abundance of Proteobacteria and 1.6-fold significantly lower Firmicutes than non-SIBO subjects. Furthermore, altered Proteobacterial profiles were found that correlated with symptom severity. Furthermore, a study by Barlow et al. (2021), evaluated the total and taxon-specific absolute microbial loads from 250 duodenal-aspirate samples in patients with a wide range of gastrointestinal conditions (including both organic and functional gastrointestinal disorders). This study found higher (but not statistically significant) total microbial loads (digital PCR-based assessment) in patients with SIBO as compared to those without SIBO. Furthermore, disruptor taxa (*Enterobacteriaceae)* were enriched in many patients classified as having SIBO and high loads of disruptors correlated with a high prevalence of severe gastrointestinal symptoms. This study concluded that SIBO diagnosis *via* microbial culture should focus on quantification of a specific group of disruptor taxa (*Enterobacteriaceae*) rather than simply the total microbial load.

#### Volatiles Produced by Gastrointestinal Microbes

Microorganisms release many different volatiles also referred to as microbial volatile organic compounds (mVOCs) (Schulz-Bohm et al., 2017). These volatiles potentially play a vital role in shaping the composition of the microbial communities. mVOCs are considered to be long-distance messengers that influence growth of other microbes while soluble substances released by bacteria are important for short distance interactions. mVOCS including hydrogen sulfide, ammonia, trimethylamine, nitric oxide, and 2-amino-acetophenone can modify the biofilm formation or dispersal or affect bacterial motility (Audrain et al., 2015). However, thus far the role of mVOCs in the context of human disease has not been widely studied. However, assessing mVOCS directly from the lumen of the gut, and from biologic samples cultured from the gut has the potential to deliver additional insights into microbe-microbe interactions within the human gastrointestinal tract.

#### Luminal Gas Sensing During Gastrointestinal Transit Utilizing Novel Capsule Technology

Some of the shortcomings of the non-invasive breath tests can be theoretically overcome by using new technologies such as the Atmo Gas Capsule*^R^* which allows to determine the concentrations of gases while the capsule transits through the lumen of the GI tract. While the relative position of the capsule in the gastrointestinal tract is known, the localization of gas production due to localized alterations of composition and density of bacteria in the gastrointestinal tract can be determined. Preliminary studies (Kalantar-Zadeh et al., 2018) provide encouraging data with good correlations of breath hydrogen and regional hydrogen patterns generated utilizing the gas-sensing capsule. In particular the capsule had vastly superior signal-to-noise ratio in response to a fermentable load than traditional breath testing. As a consequence, gas-sensing capsules might be in the future a valuable approach for “direct” assessment of microbial density and provides an opportunity to overcome the shortcomings of the established current breath tests for diagnosing SIBO. We have summarized the available diagnostic tests for SIBO diagnosis in Table 2.

**TABLE 2:** Diagnostic tests for small intestinal bacterial overgrowth (SIBO) and small intestinal dysbiosis.

	Test	Advantages	Disadvantages
1	Traditional breath tests.	Non-invasive. Low costs (minimal costs for consumables). Suitable as an office-based test.	Low sensitivity and specificity. Diagnostic thresholds remain controversial, relevance of high baseline for hydrogen and/methane remains uncertain. Optimal substrate (glucose vs. lactulose).
2	Culture based techniques.	Allows quantitation of colony forming units (CFUs). Considered the gold standard for the diagnosis of SIBO.	Invasive. Most appropriate location for aspirate (distal duodenum reachable with a gastroscope vs. jejunum reachable with radiologically place aspiration catheter) undetermined. Small bowel often does not contain fluid that can be readily aspirated. Only small proportion of microbiota can be cultured, using the traditional culture methods. Lack of consensus of diagnostic thresholds in different segments of the small intestine.
3	Culture bases techniques in combination with molecular characterization of the microbes lining the mucosa associated microbiome.	Allows better characterization of microbial communities. Potentially can tailor future treatments based upon the results of the molecular characterization (e.g., use of specific diets, probiotics, or specific antibiotics).	Additional cost. So far, this technique has not been validated but field is rapidly progressing with the development of molecular techniques. Requires specific equipment to avoid cross contamination of mucosal biopsy samples with oro-pharyngeal secretions and luminal contents. Only currently used by small number of centers with good access to microbial research facilities. Thus far widely unknown how analysis and interpretation can account for PPI use, diets and nicotine use.
4	Assessment of bacterial load, calculated utilizing qPCR measurements of the bacterial 16S rRNA gene, normalized to human beta-actin expression.	Simple and well-established technique (qPCR), but limited data in relation to SIBO diagnosis. Can be routinely done during an elective endoscopy.	Limited clinical experience and thus far no formal validation in the relation to the clinical utility.
5	Gas sensing capsules with wireless transmission of data.	Non-invasive. Gases such as hydrogen, carbon dioxide, and oxygen are measured in the lumen of the small intestine with most likely very good signal to noise ratios, compared with breath traditional breath test. Suitable as an office-based test.	If capsule is swallowed the delivery of capsule and substrate (glucose) may not occur at the same time and may require endoscopic delivery of capsule and substrate. Limited clinical experience and thus far no formal validation in the relation to the clinical utility.
6	Mucosal biopsies.	Can be incorporated into routine endoscopy. Especially useful when fluid is not readily available for aspiration from the proximal small intestine. Good concordance between results (total bacterial counts and the type of organisms) obtained using small bowel aspirate and small bowel biopsy. Targets the microbes colonizing the mucosa associated microbiome. Allows culture work to be done (aerobic and anaerobic bacteria), rapid molecular techniques as well metagenomics, metatranscriptomics, metaproteomics, metabonomics and metabolomics.	Specific biopsy technique/device required to avoid cross contamination.

## Antimicrobial Therapies Targeting Small Intestine Bacterial Overgrowth in Functional Dyspepsia Patients

Contrasting the relatively large number of studies exploring antimicrobial therapy in IBS (Ford et al., 2018), there are limited data on the effects of antimicrobial therapy in FD. A well-designed study from Hongkong comparing 2 weeks of rifaximin and placebo demonstrated the efficacy of active medication in relation to satisfactory improvement of overall dyspeptic symptoms, post-prandial fullness, belching or bloating in FD patients but breath testing, or microbiome analysis was not reported (Tan et al., 2017). On the other hand, antimicrobial therapy is frequently used in FD patients with concomitant *Helicobacter pylori* infection under the assumption that *H. pylori* is the cause of symptoms. Indeed, based upon the most recent systematic review and meta-analysis, antimicrobial therapy is superior to placebo in relation to the reduction of FD symptoms (Kang et al., 2019). On the other hand, in otherwise healthy subjects such as blood donors, and utilizing serologic *H. pylori* testing, the seroprevalence of *H. pylori* was similar among the different categories of dyspepsia and it could be concluded that infection with *H. pylori* is not associated with abdominal complaints in otherwise healthy subjects (Holtmann et al., 1994). Thus, the beneficial effects of antimicrobial therapy in FD patients with *H. pylori* infection could be at least partly mediated *via* effects on the small intestinal microbiome. Interestingly, a prospective study revealed that in FD patients, the response to antimicrobial therapy with rifaximin is not influenced by the presence of IBS symptoms (Shah et al., 2021). Furthermore, antibiotic therapy in FD patients with *H. pylori* significantly improved symptoms in a subgroup of patients (Ford et al., 2022). On the other hand, in otherwise healthy blood donors a *H. pylori* infection is not associated with dyspeptic symptoms (Holtmann et al., 1994). This points toward the possibility that symptom improvement after antibiotic therapy targeting *H. pylori* is actually not mediated by *H. pylori* but, e.g., other alterations of the gut microbiome. Altogether, the data point toward the possibility that alterations of the gastrointestinal microbiome in patients with FD and IBS can be successfully targeted with antimicrobial therapies that ultimately result in at least temporary relief of symptoms. Improvement in symptoms following treatment with antibiotics, provide evidence that dysbiosis may play a role in the pathophysiology in at least a subgroup of patients with FGIDs.

## Conclusion and Perspectives

Emerging data strongly suggest–while not undisputed–that the microbes colonizing the gastrointestinal tract are vital in the pathophysiology of FGIDs including FD. Interventions targeting the gastrointestinal microbiome thus may provide opportunities to treat and improve symptoms or potentially even cure symptoms in at least a subgroup of patients. However, progress in this field is not only hampered by the lack of appropriately designed clinical trials (e.g., relying on cross section studies and not accounting for relevant confounders) but even more importantly by the lack of simple and reliable diagnostic tests. To characterize the potential link between intestinal dysbiosis and FD it is not only required to address the methodological limitations of the traditional tests for SIBO. This includes the poor sensitivity and specificity of breath tests which are commonly used in clinical settings, or culture techniques which are invasive and restricted to microbes that can be cultured. In addition, confounders need to be taken into consideration which include–but not limited to–medications (Jones et al., 2021), infections (e.g., post-infectious FD) or parasitic infestations and their impact on the mucosal immune system (Shah et al., 2022), psychological co-morbidities (von Wulffen et al., 2019) and subsequent alterations of diet on the gastrointestinal microbiome (Saffouri et al., 2019), to define the pathophysiological links between FD and gastrointestinal dysbiosis.

In the clinical setting, glucose or lactulose breath tests are widely used. These tests are based upon the measurement of hydrogen or methane in the breath after oral ingestion of defined amounts of carbohydrate substrates. While the validity and sensitivity and specificity of the diagnostic test for SIBO rely on specific thresholds, direct culture test quantitate colony forming units when duodenal aspirates or biopsies are cultured. However, only a small proportion of microbes colonizing the small intestinal tract can be cultured. The obvious gap relates to diagnostic approaches that allow the characterization of microbes colonizing the mucosal lining of the gastrointestinal tract utilizing molecular techniques. An interesting novel approach might be the use of biosensors that are delivered *via* ingested capsules and measure concentration within the gastrointestinal tract as these capsules transit though the gastrointestinal tract. However, this indirect approach currently only captures one or two defined bacterial metabolites limiting the utility. Thus, in order to precisely characterize the small intestinal microbiome, there is an opportunity to validate and use novel molecular techniques in the routine clinical setting. It will be important to use these molecular techniques to further characterize the gut microbiome and explore the response to antimicrobial therapy including the links between long lasting symptom resolution and the microbiome. This will pave the path for novel diagnostic and therapeutic approaches that ultimately will allow to define the role of microbes for the pathophysiology of FD and potentially to individualize treatments and ultimately improve patient outcomes.

## Author Contributions

AS and GH: study idea, concept and design, drafting of the first version of the manuscript, and review of the final manuscript. NT: drafting of the manuscript and review of the final manuscript. All authors contributed to the article and approved the submitted version.

## Conflict of Interest

NT reports personal fees from Allakos, Aviro Health, Antara Life Sciences, Arlyx, Bayer, Danone, Planet Innovation, Takeda, Viscera Labs, twoXAR, Viscera Labs, Dr. Falk Pharma, Censa, Cadila Pharmaceuticals, Progenity Inc., Sanofi-aventis, Glutagen, ARENA Pharmaceuticals, IsoThrive, BluMaiden, HVN National Science Challenge, non-financial support from HVN National Science Challenge NZ, outside the submitted work. In addition, NT has a patent Biomarkers of IBS licensed (#12735358.9-1405/2710383) and (#12735358.9-1405/2710384), a patent Licensing Questionnaires Talley Bowel Disease Questionnaire licensed to Mayo/NT, a patent Nestec European Patent licensed, and a patent Singapore Provisional Patent NTU Ref: TD/129/17 “Microbiota Modulation of BDNF Tissue Repair Pathway” issued and copyright Nepean Dyspepsia Index (NDI) 1998 and Editorial: Medical Journal of Australia (Editor in Chief), Up to Date (Section Editor), Precision and Future Medicine, Sungkyunkwan University School of Medicine, South Korea, Med (Journal of Cell Press). NT participates Committees: Australian Medical Council (AMC) Council Member (2016–2019), MBS Review Taskforce (2016–2020), NHMRC Principal Committee, Research Committee (2016–2021), Asia Pacific Association of Medical Journal Editors (APAME) (current), GESA Board Member (2017–2019). NT Misc: Avant Foundation (judging of research grants) (2019). NT community and patient advocacy groups: Advisory Board, IFFGD (International Foundation for Functional GI Disorders). NT holds an NHMRC Investigator grant. GH report to be on the advisory boards Australian Biotherapeutics, Glutagen, Bayer and received research support from Bayer, Abbott, Pfizer, Janssen, Takeda, Allergan. He serves on the Boards of the West Moreton Hospital and Health Service, Queensland, UQ Healthcare, Brisbane, and the Gastro-Liga, Germany. He has a patent for the Brisbane aseptic biopsy device and serves as Editor of the Gastro-Liga Newsletter. The remaining author declares that the research was conducted in the absence of any commercial or financial relationships that could be construed as a potential conflict of interest.

## Publisher’s Note

All claims expressed in this article are solely those of the authors and do not necessarily represent those of their affiliated organizations, or those of the publisher, the editors and the reviewers. Any product that may be evaluated in this article, or claim that may be made by its manufacturer, is not guaranteed or endorsed by the publisher.

## References

[B1] Audrain B. Farag M. A. Ryu C. M. Ghigo J. M. (2015). Role of bacterial volatile compounds in bacterial biology. *FEMS Microbiol. Rev.* 39 222–233. 25725014 10.1093/femsre/fuu013

[B2] Bardhan P. K. Gyr K. Beglinger C. Vogtlin J. Frey R. Vischer W. (1992). Diagnosis of bacterial overgrowth after culturing proximal small-bowel aspirate obtained during routine upper gastrointestinal endoscopy. *Scand. J. Gastroenterol.* 27 253–256. 10.3109/00365529208999959 1502491

[B3] Barlow J. T. Leite G. Romano A. E. Sedighi R. Chang C. Celly S. (2021). Quantitative sequencing clarifies the role of disruptor taxa, oral microbiota, and strict anaerobes in the human small-intestine microbiome. *Microbiome* 9:214. 10.1186/s40168-021-01162-2 34724979 PMC8561862

[B4] Bik E. M. Eckburg P. B. Gill S. R. Nelson K. E. Purdom E. A. Francois F. (2006). Molecular analysis of the bacterial microbiota in the human stomach. *Proc. Natl. Acad. Sci. U.S.A.* 103 732–737. 10.1073/pnas.0506655103 16407106 PMC1334644

[B5] Bjorneklett A. Jenssen E. (1982). Relationships between hydrogen (H2) and methane (CH4) production in man. *Scand. J. Gastroenterol.* 17 985–992. 7167741

[B6] Bohm M. Siwiec R. M. Wo J. M. (2013). Diagnosis and management of small intestinal bacterial overgrowth. *Nutr. Clin. Pract.* 28 289–299. 10.1177/0884533613485882 23614961

[B7] Bond J. H. Jr. Levitt M. D. (1972). Use of pulmonary hydrogen (H 2) measurements to quantitate carbohydrate absorption. Study of partially gastrectomized patients. *J. Clin. Invest.* 51 1219–1225. 10.1172/JCI106916 5020434 PMC292253

[B8] Bouhnik Y. Alain S. Attar A. Flourie B. Raskine L. Sanson-Le Pors M. J. (1999). Bacterial populations contaminating the upper gut in patients with small intestinal bacterial overgrowth syndrome. *Am. J. Gastroenterol.* 94 1327–1331. 10.1111/j.1572-0241.1999.01016.x 10235214

[B9] Brophy S. Jones K. H. Rahman M. A. Zhou S. M. John A. Atkinson M. D. (2013). Incidence of Campylobacter and *Salmonella* infections following first prescription for PPI: a cohort study using routine data. *Am. J. Gastroenterol.* 108 1094–1100. 10.1038/ajg.2013.30 23588238

[B10] Camilleri M. Madsen K. Spiller R. Van Meerveld B. G. Verne G. N. (2012). Intestinal barrier function in health and gastrointestinal disease. *Neurogastroenterol. Motil.* 24 503–512. 10.1111/j.1365-2982.2012.01921.x 22583600 PMC5595063

[B11] Cangemi D. J. Lacy B. E. Wise J. (2021). Diagnosing small intestinal bacterial overgrowth: a comparison of lactulose breath tests to small bowel aspirates. *Dig. Dis. Sci.* 66 2042–2050. 10.1007/s10620-020-06484-z 32681227

[B12] Carding S. Verbeke K. Vipond D. T. Corfe B. M. Owen L. J. (2015). Dysbiosis of the gut microbiota in disease. *Microb. Ecol. Health Dis.* 26:26191. 25651997 10.3402/mehd.v26.26191 PMC4315779

[B13] Chandra S. Dutta U. Noor M. T. Taneja N. Kochhar R. Sharma M. (2010). Endoscopic jejunal biopsy culture: a simple and effective method to study jejunal microflora. *Indian J. Gastroenterol.* 29 226–230. 10.1007/s12664-010-0072-6 21210269

[B14] Christman N. T. Hamilton L. H. (1982). A new chromatographic instrument for measuring trace concentrations of breath-hydrogen. *J. Chromatogr.* 229 259–265. 10.1016/s0378-4347(00)84268-1 7096464

[B15] Corazza G. R. Menozzi M. G. Strocchi A. Rasciti L. Vaira D. Lecchini R. (1990). The diagnosis of small bowel bacterial overgrowth. Reliability of jejunal culture and inadequacy of breath hydrogen testing. *Gastroenterology* 98 302–309. 10.1016/0016-5085(90)90818-l 2295385

[B16] Cuiv O. Aguirre de Carcer D. Jones M. Klaassens E. S. Worthley D. L. Whitehall V. L. (2011). The effects from DNA extraction methods on the evaluation of microbial diversity associated with human colonic tissue. *Microb. Ecol.* 61 353–362. 10.1007/s00248-010-9771-x 21153634

[B17] Daniel H. (2022). Diet and gut microbiome and the “Chicken or Egg” problem. *Front. Nutr.* 8:828630. 10.3389/fnut.2021.828630 35178420 PMC8844458

[B18] Dominguez-Bello M. G. Costello E. K. Contreras M. Magris M. Hidalgo G. Fierer N. (2010). Delivery mode shapes the acquisition and structure of the initial microbiota across multiple body habitats in newborns. *Proc. Natl. Acad. Sci U.S.A.* 107 11971–11975. 10.1073/pnas.1002601107 20566857 PMC2900693

[B19] Drasar B. S. Shiner M. (1969). Studies on the intestinal flora. II. Bacterial flora of the small intestine in patients with gastrointestinal disorders. *Gut* 10 812–819. 10.1136/gut.10.10.812 4981709 PMC1552988

[B20] Drasar B. S. Shiner M. McLeod G. M. (1969). Studies on the intestinal flora. I. The bacterial flora of the gastrointestinal tract in healthy and achlorhydric persons. *Gastroenterology* 56 71–79. 4885396

[B21] Eckburg P. B. Bik E. M. Bernstein C. N. Purdom E. Dethlefsen L. Sargent M. (2005). Diversity of the human intestinal microbial flora. *Science* 308:1635. 10.1126/science.1110591 15831718 PMC1395357

[B22] Enck P. Azpiroz F. Boeckxstaens G. Elsenbruch S. Feinle-Bisset C. Holtmann G. (2017). Functional dyspepsia. *Nat. Rev. Dis. Primers* 3:17081. 29099093 10.1038/nrdp.2017.81

[B23] Erdogan A. Rao S. S. C. (2015). Small intestinal fungal overgrowth. *Curr. Gastroenterol. Rep.* 17:16. 25786900 10.1007/s11894-015-0436-2

[B24] Erdogan A. Lee Y. Y. Sifuentes H. Rao S. S. (2014). Sa2026 small intestinal fungal overgrowth (SIFO): a cause of gastrointestinal symptoms. *Gastroenterology* 146:358.

[B25] Fan X. Sellin J. H. (2009). Review article: small intestinal bacterial overgrowth, bile acid malabsorption and gluten intolerance as possible causes of chronic watery diarrhoea. *Aliment. Pharmacol. Ther.* 29 1069–1077. 10.1111/j.1365-2036.2009.03970.x 19222407

[B26] Ford A. C. Harris L. A. Lacy B. E. Quigley E. M. M. Moayyedi P. (2018). Systematic review with meta-analysis: the efficacy of prebiotics, probiotics, synbiotics and antibiotics in irritable bowel syndrome. *Aliment. Pharmacol. Ther.* 48 1044–1060. 10.1111/apt.15001 30294792

[B27] Ford A. C. Tsipotis E. Yuan Y. Leontiadis G. I. Moayyedi P. (2022). Efficacy of *Helicobacter* pylori eradication therapy for functional dyspepsia: updated systematic review and meta-analysis. *Gut* gutjnl-2021–326583. 10.1136/gutjnl-2021-326583 [Epub ahead of print]. 35022266

[B28] Futagami S. Itoh T. Sakamoto C. (2015). Systematic review with meta-analysis: post-infectious functional dyspepsia. *Aliment. Pharmacol. Ther.* 41 177–188. 10.1111/apt.13006 25348873

[B29] Gandhi A. Shah A. Jones M. P. Koloski N. Talley N. J. Morrison M. (2021). Methane positive small intestinal bacterial overgrowth in inflammatory bowel disease and irritable bowel syndrome: a systematic review and meta-analysis. *Gut Microbes* 13:1933313. 10.1080/19490976.2021.1933313 34190027 PMC8253120

[B30] Gasbarrini A. Corazza G. R. Gasbarrini G. Montalto M. Di Stefano M. Basilisco G. (2009). Methodology and indications of H2-breath testing in gastrointestinal diseases: the rome consensus conference. *Aliment. Pharmacol. Ther.* 29(Suppl. 1) 1–49. 10.1111/j.1365-2036.2009.03951.x 19344474

[B31] Gevers D. Kugathasan S. Denson L. A. Vazquez-Baeza Y. Van Treuren W. Ren B. (2014). The treatment-naive microbiome in new-onset Crohn’s disease. *Cell Host Microbe* 15 382–392. 24629344 10.1016/j.chom.2014.02.005 PMC4059512

[B32] Ghoshal U. C. Srivastava D. Misra A. (2018). A randomized double-blind placebo-controlled trial showing rifaximin to improve constipation by reducing methane production and accelerating colon transit: a pilot study. *Indian J. Gastroenterol.* 37 416–423. 10.1007/s12664-018-0901-6 30406392

[B33] Ghoshal U. Ghoshal U. C. Ranjan P. Naik S. R. Ayyagari A. (2003). Spectrum and antibiotic sensitivity of bacteria contaminating the upper gut in patients with malabsorption syndrome from the tropics. *BMC Gastroenterol.* 3:9. 10.1186/1471-230X-3-9 12769832 PMC165422

[B34] Gorbach S. L. Plaut A. G. Nahas L. Weinstein L. Spanknebel G. Levitan R. (1967). Studies of intestinal microflora. II. Microorganisms of the small intestine and their relations to oral and fecal flora. *Gastroenterology* 53 856–867. 4863722

[B35] Gorkiewicz G. Thallinger G. G. Trajanoski S. Lackner S. Stocker G. Hinterleitner T. (2013). Alterations in the colonic microbiota in response to osmotic diarrhea. *PLoS One* 8:e55817. 10.1371/journal.pone.0055817 23409050 PMC3568139

[B36] Grace E. Shaw C. Whelan K. Andreyev H. J. (2013). Review article: small intestinal bacterial overgrowth–prevalence, clinical features, current and developing diagnostic tests, and treatment. *Aliment. Pharmacol. Ther.* 38 674–688. 10.1111/apt.12456 23957651

[B37] Guarner F. Malagelada J. R. (2003). Gut flora in health and disease. *Lancet* 361 512–519. 10.1016/s0140-6736(03)12489-0 12583961

[B38] Gurusamy S. R. Shah A. Talley N. J. Koloski N. Jones M. P. Walker M. M. (2021). Small intestinal bacterial overgrowth in functional dyspepsia: a systematic review and meta-analysis. *Am. J. Gastroenterol.* 116 935–942. 10.14309/ajg.0000000000001197 33734110

[B39] Gutierrez I. M. Kang K. H. Calvert C. E. Johnson V. M. Zurakowski D. Kamin D. (2012). Risk factors for small bowel bacterial overgrowth and diagnostic yield of duodenal aspirates in children with intestinal failure: a retrospective review. *J. Pediatr. Surg.* 47 1150–1154. 10.1016/j.jpedsurg.2012.03.019 22703785 PMC3377944

[B40] Hamilton I. Worsley B. W. Cobden I. Cooke E. M. Shoesmith J. G. Axon A. T. (1982). Simultaneous culture of saliva and jejunal aspirate in the investigation of small bowel bacterial overgrowth. *Gut* 23 847–853. 10.1136/gut.23.10.847 6749605 PMC1419827

[B41] Hirakawa M. Iida M. Kohrogi N. Fujishima M. (1988). Hydrogen breath test assessment of orocecal transit time: comparison with barium meal study. *Am. J. Gastroenterol.* 83 1361–1363. 3195540

[B42] Holtmann G. Goebell H. Holtmann M. Talley N. J. (1994). Dyspepsia in healthy blood donors. Pattern of symptoms and association with *Helicobacter* pylori. *Dig. Dis. Sci.* 39 1090–1098. 10.1007/BF02087563 8174422

[B43] Holtmann G. Shah A. Morrison M. (2017). Pathophysiology of functional gastrointestinal disorders: a holistic overview. *Dig. Dis.* 35(Suppl. 1) 5–13. 10.1159/000485409 29421808

[B44] Imhann F. Bonder M. J. Vich Vila A. Fu J. Mujagic Z. Vork L. (2016). Proton pump inhibitors affect the gut microbiome. *Gut* 65 740–748. 10.1136/gutjnl-2015-310376 26657899 PMC4853569

[B45] Jacobs C. Coss Adame E. Attaluri A. Valestin J. Rao S. S. (2013). Dysmotility and proton pump inhibitor use are independent risk factors for small intestinal bacterial and/or fungal overgrowth. *Aliment. Pharmacol. Ther.* 37 1103–1111. 10.1111/apt.12304 23574267 PMC3764612

[B46] Jones M. P. Shah A. Walker M. M. Koloski N. A. Holtmann G. Talley N. J. (2021). Antibiotic use but not gastrointestinal infection frequently precedes first diagnosis of functional gastrointestinal disorders. *U. Eur Gastroenterol. J.* 9 1074–1080. 10.1002/ueg2.12164 34653313 PMC8598965

[B47] Kalantar-Zadeh K. Berean K. J. Ha N. Chrimes A. F. Xu K. Grando D. (2018). A human pilot trial of ingestible electronic capsules capable of sensing different gases in the gut. *Nat. Electron.* 1 79–87. 10.1038/s41928-017-0004-x

[B48] Kang S. J. Park B. Shin C. M. (2019). *Helicobacter* pylori eradication therapy for functional dyspepsia: a meta-analysis by region and H. pylori Prevalence. *J. Clin. Med.* 8:1324. 10.3390/jcm8091324 31466299 PMC6780123

[B49] Khoshini R. Dai S. C. Lezcano S. Pimentel M. (2008). A systematic review of diagnostic tests for small intestinal bacterial overgrowth. *Dig. Dis. Sci.* 53 1443–1454. 10.1007/s10620-007-0065-1 17990113

[B50] Kunkel D. Basseri R. J. Makhani M. D. Chong K. Chang C. Pimentel M. (2011). Methane on breath testing is associated with constipation: a systematic review and meta-analysis. *Dig. Dis. Sci.* 56 1612–1618. 10.1007/s10620-011-1590-5 21286935

[B51] Leite G. Morales W. Weitsman S. Celly S. Parodi G. Mathur R. (2020). The duodenal microbiome is altered in small intestinal bacterial overgrowth. *PLoS One* 15:e0234906. 10.1371/journal.pone.0234906 32645011 PMC7347122

[B52] Leon-Barua R. Gilman R. H. Rodriguez C. Bonilla J. J. Yi A. Maurtua D. (1993). Comparison of three methods to obtain upper small bowel contents for culture. *Am. J. Gastroenterol.* 88 925–928. 8503389

[B53] Levitt M. D. (1969). Production and excretion of hydrogen gas in man. *N. Engl. J. Med.* 281 122–127. 10.1056/nejm196907172810303 5790483

[B54] Levitt M. D. Furne J. K. Kuskowski M. Ruddy J. (2006). Stability of human methanogenic flora over 35 years and a review of insights obtained from breath methane measurements. *Clin. Gastroenterol. Hepatol.* 4 123–129. 10.1016/j.cgh.2005.11.006 16469670

[B55] Li G. Yang M. Zhou K. Zhang L. Tian L. Lv S. (2015). Diversity of duodenal and rectal microbiota in biopsy tissues and luminal contents in healthy volunteers. *J. Microbiol. Biotechnol.* 25 1136–1145. 10.4014/jmb.1412.12047 25737115

[B56] Lin H. C. (2004). Small intestinal bacterial overgrowth: a framework for understanding irritable bowel syndrome. *JAMA* 292 852–858. 10.1001/jama.292.7.852 15316000

[B57] Loman N. J. Pallen M. J. (2015). Twenty years of bacterial genome sequencing. *Nat. Rev. Microbiol.* 13 787–794. 10.1038/nrmicro3565 26548914

[B58] Losurdo G. Marra A. Shahini E. Girardi B. Giorgio F. Amoruso A. (2017). Small intestinal bacterial overgrowth and celiac disease: a systematic review with pooled-data analysis. *Neurogastroenterol. Motil.* 29 16–28. 10.1111/nmo.13028 28191721

[B59] Lynch S. V. Pedersen O. (2016). The human intestinal microbiome in health and disease. *N. Engl. J. Med.* 375 2369–2379. 27974040 10.1056/NEJMra1600266

[B60] Metz G. Drasar B. S. Gassull M. A. Jenkins D. J. A. Blendis L. M. (1976). Breath-hydrogen test for small-intestinal bacterial colonisation. *Lancet* 307 668–669. 10.1016/s0140-6736(76)92779-3 73641

[B61] Nobile C. J. Johnson A. D. (2015). Candida albicans biofilms and human disease. *Annu. Rev. Microbiol.* 69 71–92. 10.1146/annurev-micro-091014-104330 26488273 PMC4930275

[B62] O’Hara A. M. Shanahan F. (2006). The gut flora as a forgotten organ. *EMBO Rep.* 7 688–693. 10.1038/sj.embor.7400731 16819463 PMC1500832

[B63] Paik C. N. Choi M. G. Lim C. H. Park J. M. Chung W. C. Lee K. M. (2011). The role of small intestinal bacterial overgrowth in postgastrectomy patients. *Neurogastroenterol. Motil.* 23 e191–e196. 21324050 10.1111/j.1365-2982.2011.01686.x

[B64] Pimentel M. (2016). Breath testing for small intestinal bacterial overgrowth: should we bother? *Am. J. Gastroenterol.* 111 307–308. 10.1038/ajg.2016.30 26902227

[B65] Pimentel M. Saad R. J. Long M. D. Rao S. S. C. (2020). Clinical guideline: small intestinal bacterial overgrowth. *Am. J. Gastroenterol.* 115 165–178. 10.14309/ajg.0000000000000501 32023228

[B66] Pinto-Sanchez M. I. Yuan Y. Hassan A. Bercik P. Moayyedi P. (2017). Proton pump inhibitors for functional dyspepsia. *Cochrane Database Syst. Rev.* 11:Cd011194. 28271513 10.1002/14651858.CD011194.pub2 PMC6464600

[B67] Plaut A. G. Gorbach S. L. Nahas L. Weinstein L. Spanknebel G. Levitan R. (1967). Studies of intestinal microflora. 3. The microbial flora of human small intestinal mucosa and fluids. *Gastroenterology* 53 868–873. 6064998

[B68] Pyleris E. Giamarellos-Bourboulis E. J. Tzivras D. Koussoulas V. Barbatzas C. Pimentel M. (2012). The prevalence of overgrowth by aerobic bacteria in the small intestine by small bowel culture: relationship with irritable bowel syndrome. *Dig. Dis. Sci.* 57 1321–1329. 10.1007/s10620-012-2033-7 22262197

[B69] Quigley E. M. (2013). Gut bacteria in health and disease. *Gastroenterol. Hepatol.* 9 560–569. PMC3983973 24729765

[B70] Quigley E. M. (2014). Small intestinal bacterial overgrowth: what it is and what it is not. *Curr. Opin. Gastroenterol.* 30 141–146. 10.1097/mog.0000000000000040 24406476

[B71] Quigley E. M. Abu-Shanab A. (2010). Small intestinal bacterial overgrowth. *Infect. Dis. Clin. North Am.* 24 943–959. 20937459 10.1016/j.idc.2010.07.007

[B72] Resta S. C. (2009). Effects of probiotics and commensals on intestinal epithelial physiology: implications for nutrient handling. *J. Physiol.* 587 4169–4174. 10.1113/jphysiol.2009.176370 19596893 PMC2754357

[B73] Rezaie A. Buresi M. Lembo A. Lin H. McCallum R. Rao S. (2017). Hydrogen and methane-based breath testing in gastrointestinal disorders: the north american consensus. *Am. J. Gastroenterol.* 112 775–784. 10.1038/ajg.2017.46 28323273 PMC5418558

[B74] Rhodes J. M. Middleton P. Jewell D. P. (1979). The lactulose hydrogen breath test as a diagnostic test for small-bowel bacterial overgrowth. *Scand. J. Gastroenterol.* 14 333–336. 10.3109/00365527909179892 441681

[B75] Riordan S. M. McIver C. J. Duncombe V. M. Bolin T. D. (1995). Bacteriologic analysis of mucosal biopsy specimens for detecting small-intestinal bacterial overgrowth. *Scand. J. Gastroenterol.* 30 681–685. 10.3109/00365529509096313 7481532

[B76] Riordan S. M. McIver C. J. Wakefield D. Duncombe V. M. Thomas M. C. Bolin T. D. (2001). Small intestinal mucosal immunity and morphometry in luminal overgrowth of indigenous gut flora. *Am. J. Gastroenterol.* 96 494–500. 10.1111/j.1572-0241.2001.03533.x 11232696

[B77] Riordan S. M. McIver C. J. Walker B. M. Duncombe V. M. Bolin T. D. Thomas M. C. (1996). The lactulose breath hydrogen test and small intestinal bacterial overgrowth. *Am. J. Gastroenterol.* 91 1795–1803. 8792701

[B78] Saad R. J. Chey W. D. (2014). Breath testing for small intestinal bacterial overgrowth: maximizing test accuracy. *Clin. Gastroenterol. Hepatol.* 12 1964–1972. 10.1016/j.cgh.2013.09.055 24095975

[B79] Sadik R. Abrahamsson H. Stotzer P. O. (2003). Gender differences in gut transit shown with a newly developed radiological procedure. *Scand. J. Gastroenterol.* 38 36–42. 10.1080/00365520310000410 12608462

[B80] Saffouri G. B. Shields-Cutler R. R. Chen J. Yang Y. Lekatz H. R. Hale V. L. (2019). Small intestinal microbial dysbiosis underlies symptoms associated with functional gastrointestinal disorders. *Nat. Commun.* 10:2012. 10.1038/s41467-019-09964-7 31043597 PMC6494866

[B81] Sahakian A. B. Jee S. R. Pimentel M. (2010). Methane and the gastrointestinal tract. *Dig. Dis. Sci.* 55 2135–2143. 10.1007/s10620-009-1012-0 19830557

[B82] Schulz-Bohm K. Martín-Sánchez L. Garbeva P. (2017). Microbial volatiles: small molecules with an important role in intra- and inter-kingdom interactions. *Front. Microbiol.* 8:2484. 10.3389/fmicb.2017.02484 29312193 PMC5733050

[B83] Sekirov I. Russell S. L. Antunes L. C. M. Finlay B. B. (2010). Gut microbiota in health and disease. *Physiol. Rev.* 90 859–904. 20664075 10.1152/physrev.00045.2009

[B84] Sellin J. H. Hart R. (1992). Glucose malabsorption associated with rapid intestinal transit. *Am. J. Gastroenterol.* 87 584–589. 1595644

[B85] Sender R. Fuchs S. Milo R. (2016). Revised estimates for the number of human and bacteria cells in the body. *PLoS Biol.* 14:e1002533. 10.1371/journal.pbio.1002533 27541692 PMC4991899

[B86] Shah A. Fairlie T. Brown G. Jones M. P. Eslick G. D. Duncanson K. (2022). Duodenal eosinophils and mast cells in functional dyspepsia: a systematic review and meta-analysis of case-control studies. *Clin. Gastroenterol. Hepatol.* [Epub ahead of print]. 10.1016/j.cgh.2022.01.014 35123088

[B87] Shah A. Gurusamy S. R. Hansen T. Callaghan G. Talley N. J. Koloski N. (2021). Concomitant irritable bowel syndrome does not influence the response to antimicrobial therapy in patients with functional dyspepsia. *Dig. Dis. Sci.* 10.1007/s10620-021-07149-1 [Epub ahead of print]. 34392491

[B88] Shah A. Morrison M. Holtmann G. J. (2018). Gastroduodenal “Dysbiosis”: a new clinical entity. *Curr. Treat. Options Gastroenterol.* 16 591–604. 10.1007/s11938-018-0207-x 30421297

[B89] Shah A. Talley N. J. Jones M. Kendall B. J. Koloski N. Walker M. M. (2020a). Small intestinal bacterial overgrowth in irritable bowel syndrome: a systematic review and meta-analysis of case-control studies. *Am. J. Gastroenterol.* 115 190–201. 10.14309/ajg.0000000000000504 31913194

[B90] Shah A. Talley N. J. Koloski N. Macdonald G. A. Kendall B. J. Shanahan E. R. (2020b). Duodenal bacterial load as determined by quantitative polymerase chain reaction in asymptomatic controls, functional gastrointestinal disorders and inflammatory bowel disease. *Aliment. Pharmacol. Ther.* 52 155–167. 10.1111/apt.15786 32412673

[B91] Shanahan E. R. Shah A. Koloski N. Walker M. M. Talley N. J. Morrison M. (2018). Influence of cigarette smoking on the human duodenal mucosa-associated microbiota. *Microbiome* 6:150. 10.1186/s40168-018-0531-3 30157953 PMC6116507

[B92] Shanahan E. R. Zhong L. Talley N. J. Morrison M. Holtmann G. (2016). Characterisation of the gastrointestinal mucosa-associated microbiota: a novel technique to prevent cross-contamination during endoscopic procedures. *Aliment. Pharmacol. Ther.* 43 1186–1196. 10.1111/apt.13622 27086880

[B93] Simrén M. Stotzer P. O. (2006). Use and abuse of hydrogen breath tests. *Gut* 55 297–303. 10.1136/gut.2005.075127 16474100 PMC1856094

[B94] Sundin J. Rangel I. Fuentes S. Heikamp-de Jong I. Hultgren-Hornquist E. de Vos W. M. (2015). Altered faecal and mucosal microbial composition in post-infectious irritable bowel syndrome patients correlates with mucosal lymphocyte phenotypes and psychological distress. *Aliment. Pharmacol. Ther.* 41 342–351. 10.1111/apt.13055 25521822

[B95] Swidsinski A. Loening-Baucke V. Lochs H. Hale L. P. (2005). Spatial organization of bacterial flora in normal and inflamed intestine: a fluorescence in situ hybridization study in mice. *World J. Gastroenterol.* 11 1131–1140. 10.3748/wjg.v11.i8.1131 15754393 PMC4250702

[B96] Tabaqchali S. (1970). The pathophysiological role of small intestinal bacterial flora. *Scand. J. Gastroenterol. Suppl.* 6 139–163. 4917494

[B97] Tan V. P. Liu K. S. Lam F. Y. Hung I. F. Yuen M. F. Leung W. K. (2017). Randomised clinical trial: rifaximin versus placebo for the treatment of functional dyspepsia. *Aliment. Pharmacol. Ther.* 45 767–776. 10.1111/apt.13945 28112426

[B98] Tillman R. King C. Toskes P. (1981). “Continued experience with the xylose breath test-evidence that the small bowel culture as the gold standard for bacterial overgrowth may be tarnished,” in *Gastroenterology*, ed. Kennedy Boulevard J. F. (Philadelphia, PA: Wb Saunders Co-Elsevier Inc), 19103–12899.

[B99] von Wulffen M. Talley N. J. Hammer J. McMaster J. Rich G. Shah A. (2019). Overlap of irritable bowel syndrome and functional dyspepsia in the clinical setting: prevalence and risk factors. *Dig. Dis. Sci.* 64 480–486. 10.1007/s10620-018-5343-6 30368683

[B100] Walker M. M. Talley N. J. (2014). Review article: bacteria and pathogenesis of disease in the upper gastrointestinal tract–beyond the era of *Helicobacter* pylori. *Aliment. Pharmacol. Ther.* 39 767–779. 10.1111/apt.12666 24612362

[B101] Weitsman S. Celly S. Leite G. Mathur R. Sedighi R. Barlow G. M. (2022). Effects of proton pump inhibitors on the small bowel and stool microbiomes. *Dig. Dis. Sci.* 67 224–232. 10.1007/s10620-021-06857-y 33534012

[B102] Wu H. J. Wu E. (2012). The role of gut microbiota in immune homeostasis and autoimmunity. *Gut Microbes* 3 4–14. 10.4161/gmic.19320 22356853 PMC3337124

[B103] Yu D. Cheeseman F. Vanner S. (2011). Combined oro-caecal scintigraphy and lactulose hydrogen breath testing demonstrate that breath testing detects oro-caecal transit, not small intestinal bacterial overgrowth in patients with IBS. *Gut* 60 334–340. 10.1136/gut.2009.205476 21112950

[B104] Zhong L. Shanahan E. R. Raj A. Koloski N. A. Fletcher L. Morrison M. (2017). Dyspepsia and the microbiome: time to focus on the small intestine. *Gut* 66 1168–1169. 10.1136/gutjnl-2016-312574 27489239

[B105] Zoetendal E. G. Rajilic-Stojanovic M. de Vos W. M. (2008). High-throughput diversity and functionality analysis of the gastrointestinal tract microbiota. *Gut* 57 1605–1615. 10.1136/gut.2007.133603 18941009

